# Selective Pharmacological Activation of PPARα/δ/γ Alters the Triglyceride Composition of Fatty Liver in a Diet-Induced MASLD Mouse Model

**DOI:** 10.3390/biom16081078

**Published:** 2026-07-23

**Authors:** Takako Hishiki, Akihiro Honda, Reina Tanaka, Yukiko Minomo, Otoe Minamisawa, Tsubasa Konno, Shotaro Kamata, Waka Kamichatani, Suzumi M. Tokuoka, Yoshihiro Kita, Hideo Shindou, Isao Ishii

**Affiliations:** 1 Department of Health Chemistry, Showa Pharmaceutical University, Machida, Tokyo 194-8543, Japan; 2 Department of Lipidomics, Graduate School of Medicine, The University of Tokyo, Bunkyo, Tokyo 113-0033, Japan; 3 Department of Lipid Life Science, Japan Institute for Health Security, Shinjuku, Tokyo 162-8655, Japan; 4 Department of Medical Lipid Science, Graduate School of Medicine, The University of Tokyo, Bunkyo, Tokyo 113-0033, Japan

**Keywords:** metabolic dysfunction-associated steatohepatitis, metabolic dysfunction-associated steatotic liver disease, lipidomics, liquid chromatography–mass spectrometry, pemafibrate, peroxisome proliferator-activated receptor, pioglitazone, seladelpar, triacylglycerol

## Abstract

Various high-fat diets have been used to create animal models of metabolic dysfunction-associated steatotic liver disease (MASLD) and to evaluate the effect of various therapeutic drugs. We determined the effects of PPARα/δ/γ subtype-selective agonists (pemafibrate, seladelpar, and pioglitazone, respectively) on the hepatic triglyceride (TG) profile using LC–MS in a MASLD mouse model established by administering a high-fat/high-cholesterol/high-cholic acid (HFCC) diet combined with cyclodextrin-containing water, which is thought to induce fatty liver over a short period. The livers of mice fed the HFCC/CDX diet for four weeks exhibited an approximately fivefold increase in the summed TG LC–MS signal per unit liver weight and altered TG composition compared with normal livers of mice administered a standard diet/water. Specifically, the proportion of TG54 (TG with 54 carbon atoms) species doubled, whereas the proportion of TG52 decreased by 38%. Pemafibrate did not alter the relative total TG signal but decreased the proportion of some TG58/TG56 species and increased the proportion of some TG56/TG52 species. Seladelpar did not alter the relative total TG signal, while only slightly altering TG composition. Pioglitazone reduced the relative total TG signal by 44%, which included a decrease in the proportion of polyunsaturated fatty acid-rich TG54 and an increase in the proportion of TG58/TG56 with 1–3 unsaturated bonds. We are the first to demonstrate that selective activation of PPARα/δ/γ has different effects on the TG profile in fatty liver.

## 1. Introduction

There has been a global surge in patients with metabolic dysfunction-associated steatotic liver disease (MASLD), which begins with triglyceride (TG) accumulation in the liver. Numerous drugs targeting various molecular targets have been developed [1,2,3,4]; however, only two have been approved: resmetirom [5] and semaglutide [6]. While numerous drugs targeting various molecular pathways are currently under development, peroxisome proliferator-activated receptor (PPAR) agonists hold promise for inhibiting the early stages of MASLD—a process progressing from hepatic steatosis to lipotoxicity, inflammation, and fibrosis [2]. PPARs are nuclear receptor transcription factors comprising three subtypes—PPARα, PPARβ/δ, and PPARγ—which differ in their tissue distribution and physiological functions [7]. PPARs play a major role in regulating lipid metabolism: PPARα is mainly expressed in the liver, skeletal muscle, and heart, and induces fatty acid transport and mitochondrial/peroxisomal β-oxidation; PPARδ is expressed in most organs and drives fatty acid oxidation and energy uncoupling [8]; and PPARγ is mainly expressed in adipose tissues, and promotes adipocyte differentiation, fatty acid incorporation, and TG accumulation [9].

In a previous study [10], we used an early-stage mouse model of MASLD to systematically investigate the therapeutic effects of activating PPARα/δ/γ using clinically relevant drugs—specifically, agents that are already approved with established safety profiles or those currently undergoing clinical trials for MASLD. The agents employed were pemafibrate, seladelpar, and pioglitazone, which are selective agonists for PPARα/δ/γ, respectively [11]. Although various (genetic or dietary) animal models have been established to support drug discovery for MASLD, a model that fully captures the pathophysiological, biochemical, and histological characteristics of human MASLD (e.g., inflammation, fibrosis, and hepatocyte ballooning) remains elusive [12]. Studies have attempted to reproduce these characteristics by feeding experimental animals various high-fat diets (HFDs) that mimic Western eating habits [12,13]. We generated a mouse model of diet-induced MASLD by administering a high-fat/high-cholesterol/high-cholic acid (HFCC) diet and 1% cyclodextrin (CDX)-containing drinking water to mice [10]. Initially, we administered the HFCC diet and 2% CDX water, which Duparc et al. [14] suggested would induce symptoms of nonalcoholic steatohepatitis (NASH) within three weeks; however, extreme weight loss occurred [10]. Therefore, we reduced the water content of CDX to 1% and successfully established an early-stage MASLD-like pathology (massive TG accumulation and mild inflammation/fibrosis) in just four weeks.

Through such research, we hypothesized that the three PPAR subtypes might induce distinct patterns of TG accumulation in the fatty liver of this early-stage MASLD mouse model. The objective of this study was to systematically compare the effects of PPARα/δ/γ activation on the molecular remodeling of TG by performing lipid analysis using liquid chromatography–mass spectrometry (LC–MS) on frozen liver samples prepared in our previous study [10].

## 2. Materials and Methods

### 2.1. PPAR Agonists

Pemafibrate was kindly donated by Kowa Co., Ltd. (Tokyo, Japan). Seladelpar and pioglitazone were purchased from ChemScene (Monmouth Junction, NJ, USA) and Cayman Chemical (Ann Arbor, MI, USA), respectively.

### 2.2. Animals

C57BL/6J mice (C57BL/6JJcl) were purchased from CLEA Japan (Tokyo, Japan) and housed under the following controlled conditions: temperature 23 °C ± 1 °C, humidity 55% ± 5%, 12 h dark (8 PM to 8 AM)/12 h light cycle, and 4–5 mice housed in cages measuring 225 (width) × 338 (depth) × 140 (high) mm. The mice were fed CE-2 standard dry rodent feed (CLEA Japan) with free access to water. All experimental procedures using mice were performed in accordance with the “Guidelines for the Care and Use of Laboratory Animals (8th Edition)” issued by the U.S. National Research Council. This study was conducted with the approval of the Animal Experiment Committee of Showa Pharmaceutical University (approval numbers: P-2021-2 (validity period: April 2021–March 2024) and P-2024-4 (validity period: April 2024–March 2027)).

### 2.3. Creation of MASLD Model

Six-week-old C57BL/6J male mice were fed an HFCC [high-fat (60 kcal%)/high-cholesterol (1.25%)/high-cholic acid (0.5%); catalog number D11061901 (Research Diets, New Brunswick, NJ, USA)] diet with 1% (*w*/*v*) 2-hydroxypropyl-β-cyclodextrin (CDX; Fujifilm Wako Pure Chemical Corp., Osaka, Japan) water for four weeks to establish an early-stage MASLD model [10].

Numerous animal models have been developed for MASLD research, including genetic models, dietary models, and various combinations [1]. HFDs, high-fructose(/sucrose) diets, high-fat/high-cholesterol diets, and high-fat/high-cholesterol/high-fructose(/sucrose) diets have been widely used as diet-induced models [12,13,15]. Moreover, because methionine and choline are necessary for the synthesis of lipoproteins that circulate in the bloodstream and supply TGs throughout the body, methionine/choline-deficient diets (MCDs) have also been used to establish MASLD models, either alone or in combination with high-fat content [12,13]. Although MCDs are often superior to HFDs because they result in fatty liver and steatohepatitis associated with lobular inflammation and fibrosis over a short period, their phenotypes, including insulin resistance and inflammatory/fibrogenic gene expression, somehow differ from those observed in HFD-fed animals [13]. Therefore, it might not be considered an accurate human disease model. Because previous studies differ significantly in terms of dietary composition, administration method/duration, and animal species/sex/age, and the same experiments are rarely repeated and reproducible in multiple laboratories, it is likely that no animal model exists that can perfectly replicate human MASLD [13]. Thus, the reason for selecting the HFCC/CDX diet was that, compared with similar experiments [4,16], it was possible to induce fatty liver in inbred C57BL/6J mice over a short period without inducing significant weight changes or insulin resistance as an early-stage MASLD model [10].

### 2.4. Administration of PPAR Agonists in a MASLD Model

Food intake measurements were performed during PPAR agonist dose setting but were not continuously recorded throughout the entire treatment period in our previous study using the identical HFCC/CDX mouse model [10]. Based on the amount of food consumed, the maximum dose of each PPAR-selective agonist (0.1 mg/kg (body weight)/day for pemafibrate, 1 mg/kg/day for seladelpar, and 3 mg/kg/day for pioglitazone) that could be administered without significant weight changes and could confer selective activation of PPARα/δ/γ was determined [10]. These drugs were mixed into the HFCC feed and were ingested freely with the CDX fluid, achieving pharmacologically relevant blood drug concentrations [10]. Therefore, the present study was conducted using a previously validated fixed-dose regimen rather than a dose–response design.

Mice were randomly allocated to cages before treatment (five mice per cage; two cages per experimental group). Although medicated diets were supplied on a cage basis, all histological and lipid analyses were performed using independently collected liver samples from individual animals. Therefore, the individual mouse was considered the experimental unit for all statistical analyses.

### 2.5. Lipid Analysis

All liver specimens used for lipid analysis were obtained from the same animal experiment reported in our previous study [10]. Livers were quickly removed from isoflurane-anesthetized mice, flash-frozen in liquid nitrogen, and stored at −80 °C until lipid extraction without repeated freeze–thaw cycles. Lipid analyses were performed using coded samples to minimize analytical bias with respect to treatment allocation. A portion of frozen liver (20.6–30.8 mg) was transferred to a specific tube containing a metal grinder (Tokken Inc., Chiba, Japan) pre-cooled with liquid nitrogen. The frozen liver tissue was pulverized into a fine powder using a TK-AM7 AUTOMILL freeze grinding device (Tokken) [17]. The sample was then mixed with 0.8 mL of 2-propanol and stirred at 4 °C for 1 h using a rotator. The metal grinder was removed, and the tube was centrifuged at 20,000× *g* for 10 min at 4 °C. Finally, 0.65 mL of the supernatant was collected and stored at −80 °C until LC–MS analysis. The lipid samples were diluted 50-fold with 2-propanol just before the analysis.

TG analysis was performed using an LC–MS-8060NX triple quadrupole mass spectrometer (Shimadzu Corporation, Kyoto, Japan) equipped with a Nexera UHPLC (Shimadzu Corporation), with some modifications to the previously described method [18]. System control, data acquisition, and data evaluation were carried out using Shimadzu LabSolutions LCMS software version 5.9. Reversed-phase chromatography was performed using a Shimadzu Shim-pack Velox C18 column (2.1 × 50 mm, 2.7 µm). The injection volume was 1 µL, the column temperature was 45 °C, and the flow rate was 0.4 mL/min. Mobile phase A consisted of 20 mM NH_4_HCO_2_/water, and mobile phase B consisted of 20% acetonitrile and 80% 2-propanol. The pump gradient program [time (%B/%A)] was as follows: 0 min (85/15), 0.1 min (85/15), 8.4 min (95/5), 9 min (95/5), 9.1 min (85/15), and 11 min (85/15). The mass spectrometer parameters were as follows: 2.5 L/min for nitrogen gas (as a nebulizer), 10 L/min drying gas, 400 °C heat block, and 250 °C desolvation line. Argon gas was used for collision-induced dissociation. The multiple reaction monitoring (MRM) transitions, optimized based on preliminary experiments, were used to target the major TG species in the mouse liver [18]. In the transition, Q1 was defined as the parent ion of the ammonium adduct, and Q3 was as the product ion generated by neutrality loss (NL) of a fatty acyl chain. Therefore, the fatty acyl chain component of the TGs can be observed along with its respective NL ion (Supplementary Materials Data Source File worksheet 1). The names of the TGs were expressed as the sum of the carbon atoms and double bond equivalents of the three fatty acyl chains. Peak areas were calculated using TRACES software [19]. The TG carbon-number group signals were calculated as the sum of the MRM signals with the identical Q1 transition. The proportion of each TG type was expressed as parts per million (ppm) of the relative total TG signals (the sum of peak areas for all TG MRM signals). The LC–MS analysis was not intended to provide absolute quantification of total hepatic TG content.

No internal standard was used for the present targeted TG analysis. All liver samples were extracted using an identical protocol and analyzed consecutively under identical LC–MS conditions within a single analytical batch. To monitor analytical performance, human serum pool (Cosmo Bio, Tokyo, Japan) was analyzed before, during, and after the analytical batch using the same LC–MS panel. These technical QC measurements confirmed stable instrument performance throughout the analytical sequence. Therefore, batch correction was not performed.

### 2.6. Statistical Analysis

Data are expressed as the mean ± standard deviation (SD) (*n*: sample size). Individual statistical comparisons of the signals for each specific summed species were performed using Prism 9.5.1 (528) software (GraphPad, San Diego, CA, USA). Unpaired two-tailed Student *t*-test was used for comparisons between two groups, and a one-way ANOVA with Tukey’s multiple comparison test was used for comparisons between five groups. A *p*-value < 0.05 was considered statistically significant.

## 3. Results

### 3.1. Effects of PPARα/δ/γ-Selective Activation on Hepatic TG and Fibrosis Levels

**Figure 1:**
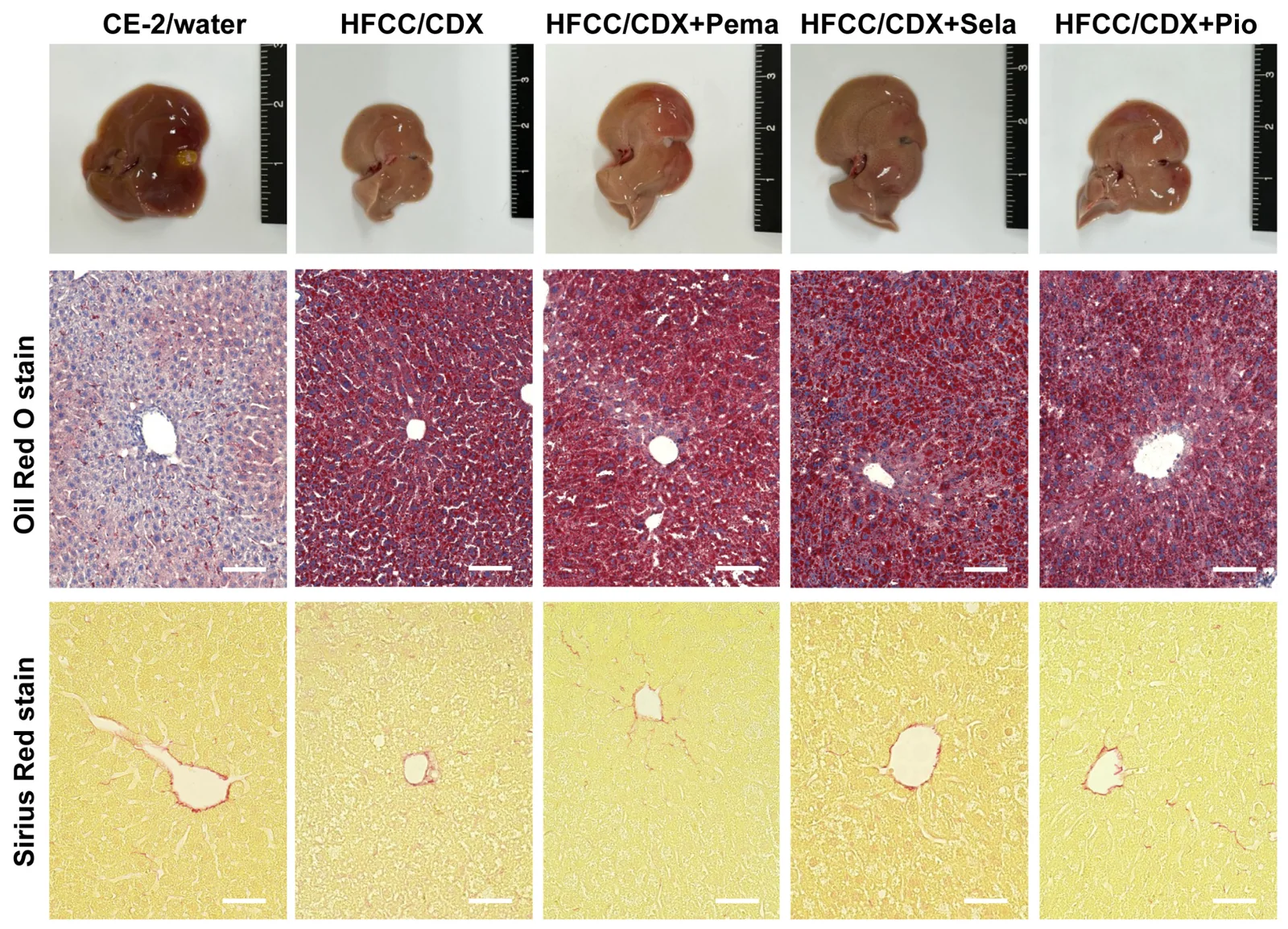
Fatty liver induced after 4 weeks of HFCC/CDX administration and the effects of concomitant treatment with pemafibrate (Pema), seladelpar (Sela), or pioglitazone (Pio). Representative macroscopic findings (top panels; cm scales), hematoxylin–Oil Red O-stained sections (middle panels), and Sirius Red-stained sections (bottom panels) of the liver of mice fed a standard CE-2 diet/tap water or HFCC/CDX for 4 weeks are shown. The HFCC/CDX groups were divided into those receiving 0.1 mg Pema/kg/day, 1 mg Sela/kg/day, or 3 mg Pio/kg/day, and those not receiving any treatment. Bar: 100 µm.

To determine the molecular changes in TGs preceding hepatic lipotoxicity/dysfunction, we created an early-stage MASLD model in which 6-week-old mice were fed an HFCC/CDX diet for 4 weeks, which resulted in significant TG accumulation (revealed by Oil Red O staining), but mild/limited fibrosis (revealed by Sirius Red staining) (Figure 1) [10]. During the same period, pemafibrate, seladelpar, and pioglitazone were added to the feed at 0.1, 1, and 3 mg/kg/day, respectively. These amounts did not have a significant effect on changes in body weight/liver weight and did not induce hepatocyte damage as evidenced by normal serum alanine/aspartate aminotransferase levels [10]. Histological examination of liver tissue sections revealed no significant changes in the summed TG LC–MS signal or fibrosis following the administration of pemafibrate, seladelpar, or pioglitazone (Figure 1).

### 3.2. LC–MS Analysis of TG Species in the MASLD Model

#### 3.2.1. TG Profiling of Fatty Liver in Mice Administered HFCC/CDX

Although the composition of a standard CE-2 diet is not publicly available, its fat content has been reported to be 4.33% (average value of the shipped products in 2025, Supplementary Materials Table S1). On the other hand, the HFCC diet contains 10.0% soy bean oil [rich in polyunsaturated fatty acids (PUFAs), such as fatty acid (FA)18:2 (linoleic acid; 50.9% of fat according to USDA FoodData Central Food Details (Supplementary Materials Table S2)) and FA18:3 (α-linolenic acid; 6.6%)], 15.1% cocoa butter [rich in monounsaturated fatty acids (MUFAs), such as FA18:1 (oleic acid: 32.6%) and FA16:1 (palmitoleic acid; 0.2%)], and 7.0% coconut oil [rich in FA4:0–24:0 saturated fatty acids (SFAs)], with an estimated total fat content of 32% (Tables S1 and S2). HFCC also contains 0.584% sodium cholate, which may promote intestinal cholesterol absorption, increase plasma VLDL/LDL cholesterol levels, and decrease plasma HDL cholesterol levels [20]. CDX has a hydrophobic cavity within its cyclic structure that increases the solubility of cholate and cholesterol by forming an inclusion complex, which promotes intestinal absorption and hepatic deposition [16]. The combination of the HFCC diet and CDX beverage was first proposed by Duparc et al. to establish a dietary model of nonalcoholic steatohepatitis (NASH) over a short period of time [14].

**Figure 2:**
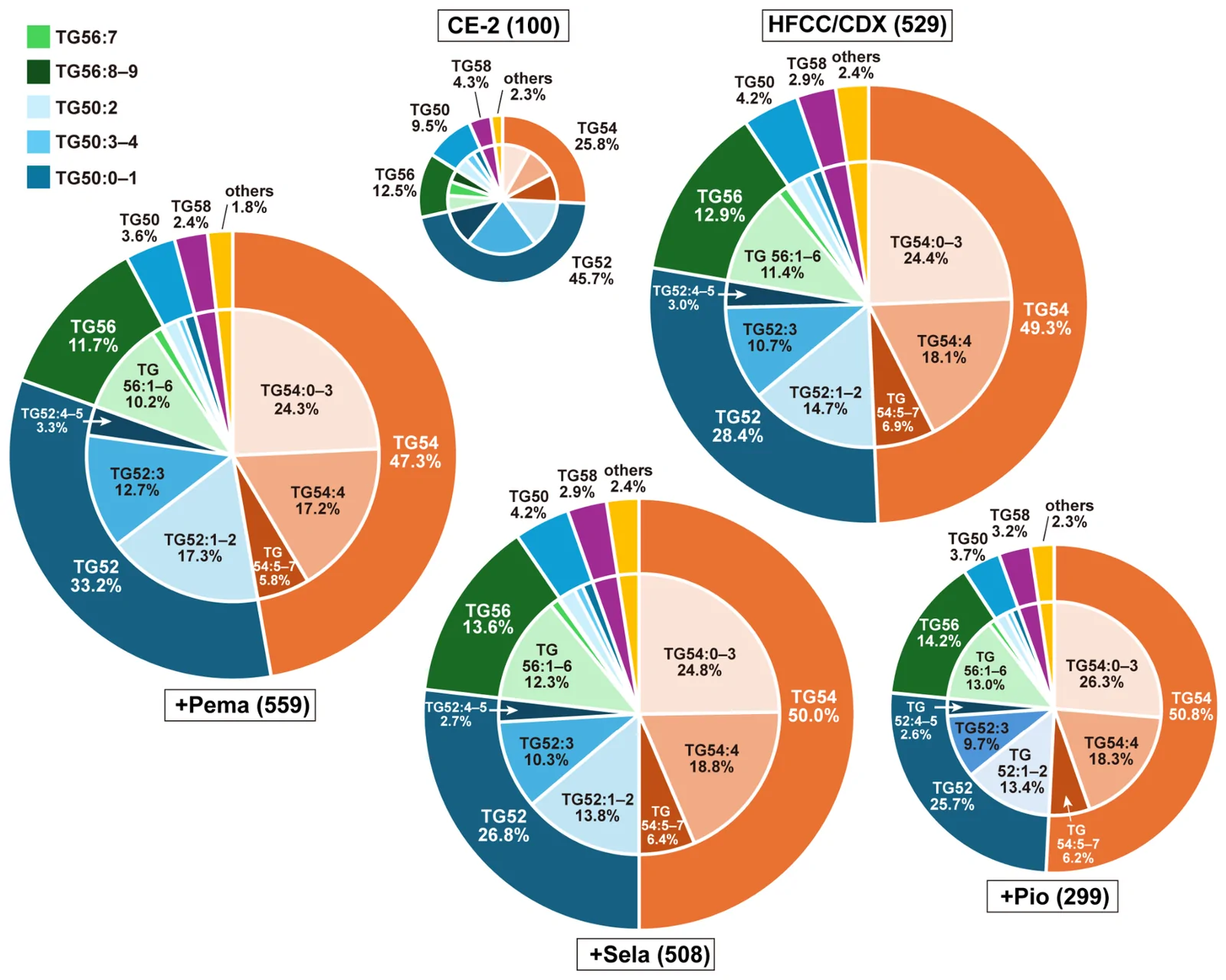
TG composition of fatty liver induced by 4 weeks of HFCC/CDX administration and the effects of concomitant administration with pemafibrate (Pema), seladelpar (Sela), or pioglitazone (Pio). LC–MS lipidomic analysis of livers of mice fed either a standard CE-2 diet or HFCC/CDX for 4 weeks with or without each PPARα/δ/γ-selective agonist (0.1 mg Pema/kg/day, 1 mg Sela/kg/day, or 3 mg Pio/kg/day). Summed liver TG signals for each group are shown as relative total signal levels with the CE-2 control set to 100, and the amount of each TG signal is expressed as a percentage, with the total TG signal in each group set at 100%.

Our lipid TG analyses using LC–MS/MS detected a total of 225 different MRM signals (Data Source File worksheets 2 and 3). Administration of HFCC/CDX resulted in a > 5-fold increase in the relative total hepatic TG signal within four weeks (Figure 2). The proportion of TG54 nearly doubled with a significant decrease in the proportions of TG52, TG50, and TG58, while the proportions of TG56 and the others remained unchanged (Data Source File worksheet 4 and Figure 2). Among the TG54 species, a significant increase in the proportion of TG54:0–4 species was observed (Figure 2).

#### 3.2.2. Effects of Pemafibrate on TG Components

**Figure 3:**
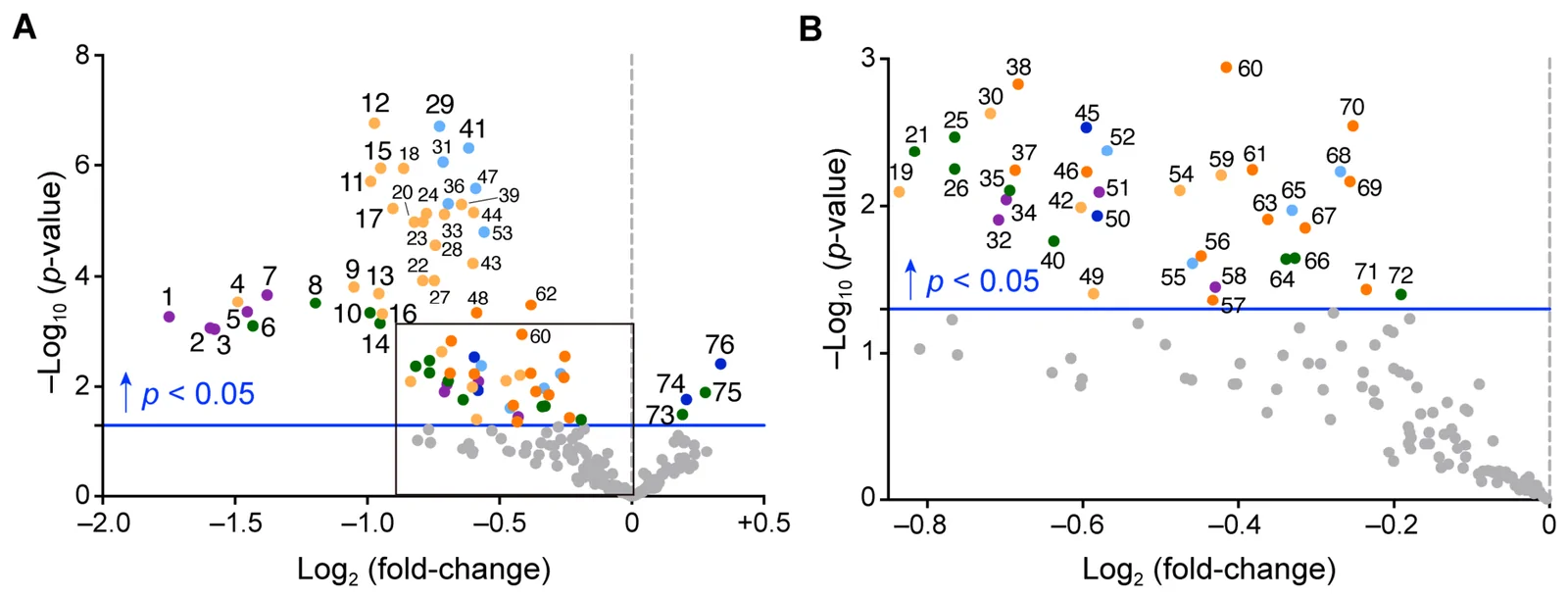
Volcano plot analysis of individual TG changes following pemafibrate administration. (**B**) is an enlarged view of a part of (**A**) (the rectangular frame). Fold-change and *p*-values were calculated for all TGs detected in the livers of mice administered HFCC/CDX without and with 0.1 mg/kg/day pemafibrate (*n* = 10 and 9, respectively). The *x*-axis represents Log_2_ (fold-change), and the *y*-axis represents −Log_10_ (*p*-value), with the *p*-value threshold set to 0.05 (blue line). The color of each dot corresponds to the major classification in Figure 2. The individual IDs and values of TGs 1–76 that exhibited significant changes are listed in Table 1.

The administration of pemafibrate (0.1 mg/kg/day) had no significant effect on the relative total TG signal; however, the proportions of TG54, TG56, TG50, and TG58 decreased slightly, whereas the proportion of TG52 increased by 17% (Figure 2). A total of 72 TG species were significantly downregulated, while only four species were upregulated (Figure 3 and Table 1). Notably, five types of TG58:1–3 (IDs: 1, 2, 3, 5, and 7) were remarkably (70–62% down) downregulated with pemafibrate treatment. Furthermore, although the 29 TG species with fewer than 50 carbon atoms quantitatively account for only 2.4% or 1.8% of all TGs, 24 of them exhibited a significant decrease, albeit to varying degrees (61–28% down) (Figure 2 and Table 1).

#### 3.2.3. Effects of Seladelpar on TG Components

**Figure 4:**
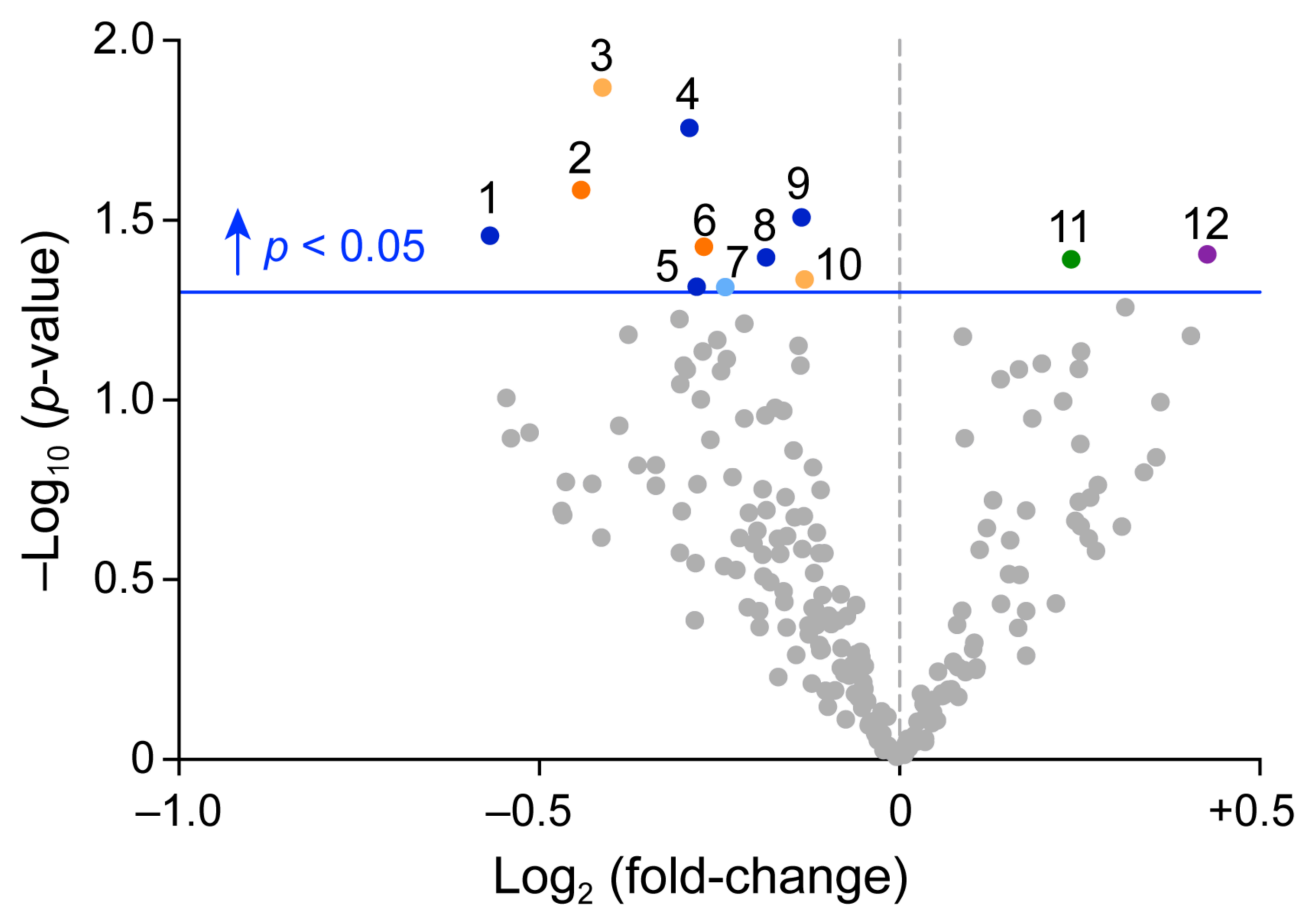
Volcano plot analysis of individual TG changes following after seladelpar administration. Fold-change and *p*-values were calculated for all TGs detected in the livers of mice administered HFCC/CDX with and without 1 mg/kg/day seladelpar (*n* = 10 each). The *x*-axis represents Log_2_ (fold-change), and the *y*-axis represents −Log_10_ (*p*-value) with the *p*-value threshold set to 0.05 (blue line). The color of each dot corresponds to the major classification in Figure 2. The individual IDs and values of TGs 1–12 showing significant changes are listed in Table 2.

Seladelpar (1 mg/kg/day) administration showed no significant effect on the relative total TG signal and TG components (Figure 2). Although the proportion of the 10 types of TG species (TG50–54) with unsaturation degrees of 1–5 decreased by 9–33%, only the proportions of TG56:4-FA20:1 and TG58:3-FA18:1 increased by 18% and 34%, respectively (Figure 4 and Table 2).

#### 3.2.4. Effects of Pioglitazone on TG Components

**Figure 5:**
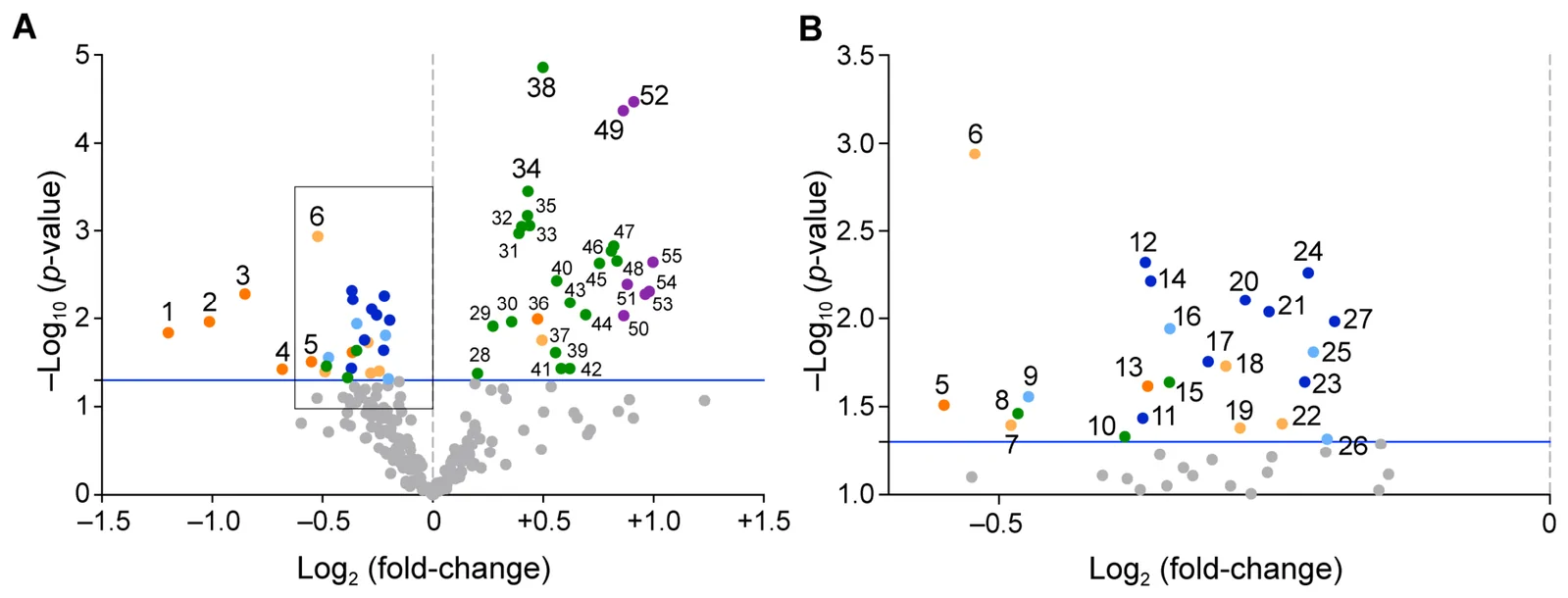
Volcano plot analysis of individual TG changes following pioglitazone administration. (**B**) is an enlarged view of a part of (**A**) (the rectangular frame). Fold changes and *p*-values were calculated for all TGs detected in the livers of mice administered HFCC/CDX with and without 3 mg/kg/day pioglitazone (*n* = 10 each). The *x*-axis represents Log_2_ (fold-change), and the *y*-axis represents −Log_10_ (*p*-value) with the *p*-value threshold set to 0.05 (blue line). The color of each dot corresponds to the major classification in Figure 2. The individual IDs and values of TGs 1–55 showing significant changes are listed in Table 3.

In contrast to pemafibrate and seladelpar, the administration of pioglitazone (3 mg/kg/day) significantly decreased the relative total TG signal by 44% without causing significant changes in the TG classification subcategory (Figure 2); however, the proportion of 55 TG species out of a total of 225 TG species decreased or increased (Figure 5 and Table 3), and these changes were characteristic of each subcategory in Figure 2. Namely, the five types of TG54:5–7 (IDs: 1–5) exhibited the largest decrease (56–32% down), followed by the nine types of TG52 (IDs: 11, 12, 14, 17, 20, 21, 23, 24, and 27) (23–13% down). In contrast, the 3 and 19 different TG56 species showed a significant decrease and increase, respectively (Figure 5 and Table 3). In particular, the 82–100% increase in seven types of TG58:1–3 (ID: 49–55) was significantly different from that observed with pemafibrate.

## 4. Discussion

This is the first study to examine how PPARα/δ/γ-selective agonists (pemafibrate, seladelpar, and pioglitazone) exert similar or different effects on the TG composition of fatty liver in a MASLD mouse model. Because PPAR agonists are expected to treat patients in the early stages of MASLD [2], we established a model by allowing mice to freely consume an HFCC/CDX diet for only four weeks (Figure 1) [10]. The HFCC/CDX model combines several dietary factors, including high fat, cholesterol, cholic acid, and cyclodextrin, each of which may influence hepatic lipid metabolism. Because the present study did not include control groups lacking individual dietary components, the specific contribution of each component to TG54 enrichment or drug-induced TG remodeling cannot be determined. Future studies using simplified dietary models or systematic comparisons of individual dietary components will be required to clarify the respective roles of these dietary factors.

Of the PPAR agonists that are expected to treat early-stage MASLD, similar to resmetirom (but different from semaglutide) [2], the pan agonist lanifibranor is the most advanced in clinical trials, followed by the α/γ dual agonist saroglitazar [4,21,22]. In addition, two phase 2 clinical trials have been initiated in Japan: a pemafibrate monotherapy trial (PRESEMT trial, NCT06623539) for non-alcoholic fatty liver disease (NAFLD) complicated with hypertriglyceridemia, and a combination therapy trial (NCT05327127) with pemafibrate and CSG452 (an SGLT2 inhibitor) for non-alcoholic steatohepatitis (NASH) with liver fibrosis [23]. Meanwhile, clinical trials involving seladelpar for NAFLD/NASH patients are currently suspended whereas trials involving pioglitazone have been ongoing for a decade [11]. Nevertheless, the effects of these drugs on the early progression of hepatic steatosis (e.g., before the diagnosis of MASLD) have not been thoroughly studied, either experimentally or clinically. Therefore, we determined how the activation of PPARα/δ/γ using approved selective agonists affects fatty liver formation in an MASLD model by comparing the hepatic TG composition.

When HFCC diets containing high concentrations of three lipid sources rich in SFAs, MUFAs, and PUFAs, respectively (Tables S1 and S2), were administered with CDX-containing drinking water, the composition of the TG54:0–4 group markedly increased (from 17.2% with CE-2/water to 42.5%; see Data Source File worksheet 4 and Figure 2). TG54:3_FA18:1 (oleic acid) appears to be the most abundant, and it could be one of the following molecules: TG54:3_FA18:1 × 3 (three FA18:1 are bound to *sn*-1–3 in the glycerol skeleton), TG54:3_FA18:1-FA16:0-FA20:2 (three different FAs are bound to *sn*-1–3), TG54:3_FA18:1-FA16:1-FA20:1, or TG54:3_FA18:1-FA18:0-FA18:2 (TG54:3_FA18:1-FA16:2-FA20:0 was not detected; Data Source File worksheets 2 and 3). One limitation of the lipid analysis was that, since we detected diacylglycerol species as fragment ions derived from TG ammonium adduct ions (parent ions [M + NH^4^]^+^) (Data Source File worksheet 1), it is unclear which FAs (except mentioned such as TG54:3_FA18:1) are attached to which positions of the glycerol, and which *sn* positions are prone to losing FAs; however, TG54:3_FA18:1, TG54:3_FA18:0, and TG54:3_FA18:2, which have high composition ratios, exhibited similar composition ratio patterns for each feed, whereas the TG54:3_FA16:0, TG54:3_FA16:1, TG54:3_FA20:1, and TG54:3_FA20:2, which have lower composition ratios, exhibited different patterns for each feed (Supplementary Materials Figure S1). These results suggest that there are specific locations within the TG molecule from which FAs are readily lost, and FA18:1, which is rich in all three lipid sources, was the major component of TG54:3. This supports the validity of our experimental system, which calculates the relative total TG signal by accumulating the signal values of each TG species (for which absolute quantification is not possible because of the lack of available TG standard substances). It is difficult to imagine that the TG composition of the liver of someone who frequently eats fatty meats would resemble that of such meat; however, this animal experiment suggests that it may be possible. In a recent 16-week randomized, parallel-group intervention trial involving 84 adults aged 22–36, the group that consumed nut snacks rich in MUFAs daily had higher plasma concentrations of MUFAs (present in the forms of phospholipids, diacylglycerols, TGs, cholesterol esters, and free FAs), as well as higher oleic acid content in their abdominal subcutaneous fat, compared to the control group that consumed carbohydrate snacks daily [24].

The present lipid analysis was designed to compare the relative abundance of TG molecular species among experimental groups. Because no internal standard was included, absolute correction for extraction efficiency and analytical variability was not possible. Nevertheless, all samples were analyzed under identical experimental conditions within a single analytical batch, and analytical stability was monitored using human serum pool analyzed throughout the measurement sequence. These procedures minimized technical variation; however, the lack of study-specific internal standards should be recognized as a limitation of the present study.

**Figure 6:**
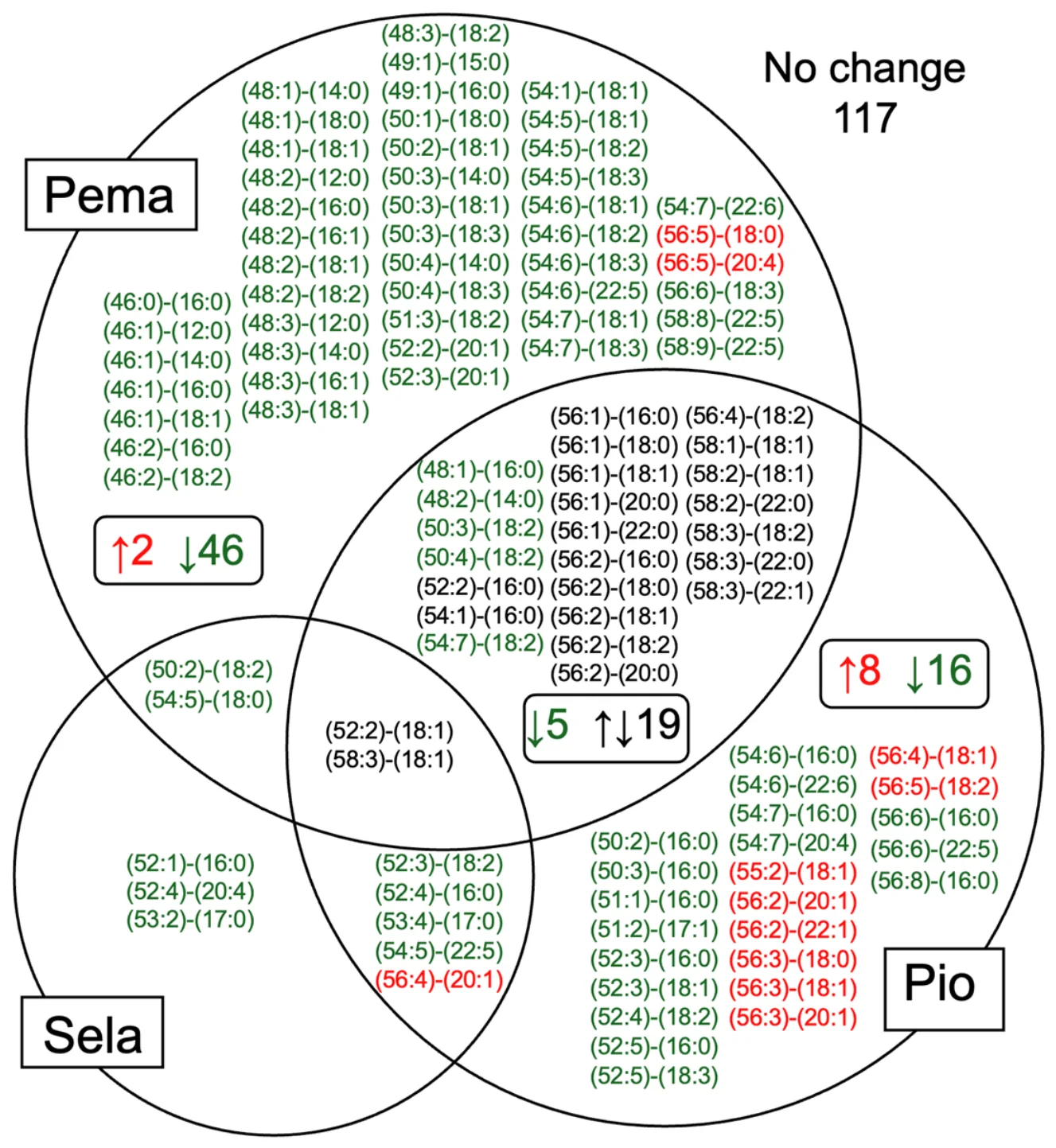
Venn diagram showing the TG species exhibiting a significant decrease or increase among all 225 detected TG species following PPAR agonist administration. For each PPAR agonist, changes are indicated in green if the value decreased and red if it increased. When changes occurred in response to multiple stimuli, changes in the same directions are shown in green or red, whereas changes in different directions are shown in black.

Figure 6 summarizes the results of classifying the 225 detected TG signals. Of these, the composition ratio of 76 TGs changed with pemafibrate stimulation, 12 TGs with seladelpar stimulation, and 55 TGs with pioglitazone stimulation (117 TGs remained unchanged). Of the three subtypes, PPARα is the primary regulator of hepatic lipid metabolism, and its agonists, known as fibrates, are used for decades to treat hypertriglyceridemia and hypercholesterolemia by reducing blood TG levels and increasing blood HDL-cholesterol levels. However, conventional fibrates (renal metabolism type) do not show efficacy against NAFLD, and in fact, they have side effects, such as renal dysfunction, which limits their effectiveness [9]. Recently, a next-generation fibrate pemafibrate (hepatic metabolism type) was developed as a selective PPARα modulator (SPPARMα) [25,26] containing a distinctive Y-shaped structure unlike other fibrates. It firmly binds to the PPARα ligand-binding site, and exhibits high PPARα selectivity/efficacy [27]. In this HFCC/CDX-induced MASLD model, pemafibrate treatment reduced serum TG levels without altering liver TG levels and Oil Red O-stained lipid droplets in the liver sections [10]. Consistently, pemafibrate did not superficially improve fatty liver (Figure 1) nor alter the relative total liver TG signal (Figure 2). However, unexpectedly, it significantly decreased the composition ratios of 72 TG species (most of which were present in small proportions), while increasing those of only 4 TG species (Figure 3 and Table 1). PPARα is physiologically activated upon fasting, perhaps by endogenous FAs released from TG stores [27]. Pemafibrate induces the expression of peroxisomal acyl-coenzyme A oxidase 1 (Acox1), enoyl-CoA hydratase, (short-chain, middle-chain, long-chain, and very long-chain) acyl-CoA dehydrogenase, as well as carnitine palmitoyl-CoA transferases 1a/2 (the components of peroxisomal and mitochondrial β-oxidation) in mouse liver in a PPARα-dependent manner [28]. Notably, the activity of pemafibrate, a TG-lowering drug, begins with the breakdown of such minor TG species.

Seladelpar did not significantly alter the relative total liver TG signal and their components (Figure 4 and Table 3). PPARδ agonists are promising drugs that combine the lipid metabolism-regulating effects of PPARα with the glucose metabolism-regulating effects of PPARγ [7], although studies remained limited compared with PPARα/γ. Consequently, the development of PPARδ agonists has been delayed, and the only approved PPARδ-selective agonist is seladelpar, which was only approved for use in primary biliary cholangitis in August 2024 following the results of the RESPONSE trial (NCT04620733) [29]. Seladelpar (1 mg/kg/day) maintained blood concentrations around the EC_50_ required for PPARδ activation observed in vitro, even after 4 weeks of administration [10], but this may be too low to significantly alter the quantity and quality of hepatic TGs.

Unlike the other two drugs, pioglitazone significantly reduces the relative total liver TG signal, resulting in a “balanced” change in the composition of 55 different TG species (27 decreased, 28 increased). Of these, there was a variety of effects in which some showed the same changes as those following pemafibrate or seladelpar administration, whereas some caused different changes, and some were unaffected (Figure 6). For cases in which the ratio increased following pioglitazone administration, most of the increases were induced specifically by pioglitazone stimulation. Of the 26 species that showed significant changes following pemafibrate and pioglitazone administration, 11 exhibited different directions of change, suggesting the opposing roles of PPARα and PPARγ. In our previous study, quantitative analyses of Oil Red O- and Sirius Red-positive areas in the liver sections revealed no statistically significant alteration among the treatment groups (except for reduced fibrosis upon pioglitazone treatment) [10]. Oil Red O staining and LC–MS evaluate different aspects of hepatic lipid accumulation. Oil Red O is a semi-quantitative histological method reflecting lipid droplet area within selected tissue sections, whereas LC–MS provides highly sensitive quantitative measurement of TG molecular species extracted from selected liver aliquots. Therefore, moderate changes in relative total liver TG signal or molecular composition may not necessarily be reflected by detectable differences in histological lipid staining.

Another limitation of this study is that the relationship between the observed changes in TG composition and the pathology/improvement of fatty liver in the MASLD model remains completely unknown. A meta-analysis of 23 studies revealed that oleic acid supplements improve blood lipid profiles [30]. Another meta-analysis of 26 randomized clinical trials involving 1244 participants also showed that dietary interventions with high-oleic acid diets slightly improve blood lipid profiles [31]. Therefore, it is unlikely that the massive hepatic accumulation of oleic acid (as TG forms), which makes up 70–80% of the components of olive oil—a heart of the Mediterranean diet—and is a ligand for PPARα/δ [27,32] and an activator of sirtuin 1 [33], and is responsible for a variety of physiological effects such as anti-inflammatory activity [34], would directly lead to the pathogenesis of MASLD. As another limitation, while we confirmed the specific induction of *Acox1* as evidence of PPARα-selective activation by pemafibrate [10], the similar PPARδ/γ-specific target engagement effects of seladelpar and pioglitazone, respectively, remain to be demonstrated in future studies.

## 5. Conclusions

Using a MASLD mouse model, we clarified the similarities and differences in the effects of three approved PPARα/δ/γ-selective agonists on the TG profile of fatty liver. All three drugs are either currently in clinical trials for MASLD or expected to see a resumption of such trials; consequently, studies investigating their effects on the liver—even those involving animal models—are highly relevant. These findings provide new insight into PPAR subtype-specific regulation of hepatic TG remodeling and may facilitate the development of PPAR-targeted therapies for MASLD and other metabolic diseases.

## Figures and Tables

**Table 1:** Changes in liver TGs following treatment with pemafibrate (Pema) in a HFCC/CDX-induced MASLD model. The TG species showing a significant decrease or increase (negative or positive in the *x*-axis) with Pema administration are listed in order of fold-change using the same ID as in Figure 2. The log_2_ (fold-change), −log_10_ (*p*-value), *p*-value, and mean ppm for HFCC/CDX (without Pema; *n* = 10) and + Pema (*n* = 9) are shown.

ID	Lipid Species	log_2_ (Fold-Change)	−log_10_ (*p*-Value)	*p*-Value	HFCC/CDX ppm	+Pema ppm
1	TG_58:3-FA_22:0	−1.7492	3.2650	0.0005433	33,025	9824
2	TG_58:2-FA_22:0	−1.5937	3.0616	0.0008678	43,956	14,564
3	TG_58:3-FA_18:2	−1.5766	3.0377	0.0009169	33,870	11,355
4	TG_46:1-FA_14:0	−1.4895	3.5374	0.0002901	7050	2511
5	TG_58:2-FA_18:1	−1.4539	3.3501	0.0004466	90,033	32,865
6	TG_56:1-FA_22:0	−1.4323	3.0975	0.0007990	4951	1835
7	TG_58:1-FA_18:1	−1.3784	3.6706	0.0002135	8731	3358
8	TG_56:1-FA_16:0	−1.1959	3.5177	0.0003036	6403	2795
9	TG_46:2-FA_16:0	−1.0504	3.8117	0.0001543	18,926	9138
10	TG_56:2-FA_18:2	−0.98978	3.3361	0.0004612	10,641	5358
11	TG_48:3-FA_18:1	−0.98716	5.7091	0.0000020	86,206	43,488
12	TG_46:2-FA_18:2	−0.97309	6.7754	0.0000002	17,536	8933
13	TG_48:3-FA_14:0	−0.95567	3.6936	0.0002025	7042	3631
14	TG_56:1-FA_18:1	−0.95135	3.1461	0.0007144	19,895	10,288
15	TG_48:3-FA_18:2	−0.94890	5.9486	0.0000011	85,413	44,246
16	TG_49:1-FA_15:0	−0.94262	3.3123	0.0004871	7393	3846
17	TG_48:3-FA_12:0	−0.90323	5.2238	0.0000060	88,334	47,231
18	TG_46:1-FA_18:1	−0.86247	5.9445	0.0000011	37,717	20,745
19	TG_48:3-FA_16:1	−0.83606	2.0963	0.0080121	6939	3887
20	TG_46:1-FA_16:0	−0.82222	4.9765	0.0000106	32,471	18,365
21	TG_56:2-FA_20:0	−0.81631	2.3705	0.0042611	322,931	183,390
22	TG_46:1-FA_12:0	−0.79015	3.9299	0.0001175	38,466	22,244
23	TG_48:2-FA_18:1	−0.78898	4.9831	0.0000104	271,245	156,984
24	TG_48:2-FA_12:0	−0.77631	5.1336	0.0000074	137,004	79,991
25	TG_56:2-FA_18:1	−0.76481	2.4706	0.0033841	577,543	339,903
26	TG_56:1-FA_18:0	−0.76467	2.2498	0.0056265	22,721	13,373
27	TG_48:2-FA_18:2	−0.74577	3.9291	0.0001177	28,512	17,003
28	TG_48:2-FA_16:0	−0.74289	4.5636	0.0000273	33,868	20,237
29	TG_50:4-FA_18:3	−0.72626	6.7175	0.0000002	14,917	9017
30	TG_48:2-FA_16:1	−0.71879	2.6311	0.0023384	25,464	15,472
31	TG_50:4-FA_14:0	−0.71353	6.0566	0.0000009	32,576	19,865
32	TG_58:3-FA_22:1	−0.70831	1.9066	0.0123990	59,155	36,205
33	TG_48:2-FA_14:0	−0.70821	5.1167	0.0000076	37,639	23,038
34	TG_58:3-FA_18:1	−0.69838	2.0438	0.0090400	136,203	83,937
35	TG_56:1-FA_20:0	−0.69389	2.1048	0.0078557	19,380	11,980
36	TG_50:4-FA_18:2	−0.69367	5.3089	0.0000049	51,104	31,597
37	TG_54:1-FA_16:0	−0.68689	2.2411	0.0057393	48,439	30,090
38	TG_54:7-FA_18:1	−0.68294	2.8290	0.0014825	13,771	8578
39	TG_48:1-FA_16:0	−0.64344	5.2948	0.0000051	101,810	65,177
40	TG_56:2-FA_16:0	−0.63716	1.7642	0.0172090	12,981	8347
41	TG_50:3-FA_18:1	−0.61613	6.3260	0.0000005	206,224	134,545
42	TG_48:1-FA_18:0	−0.60253	1.9912	0.0102040	16,128	10,622
43	TG_48:1-FA_14:0	−0.60037	4.2380	0.0000578	69,034	45,534
44	TG_48:1-FA_18:1	−0.59749	5.1506	0.0000071	80,008	52,878
45	TG_52:2-FA_20:1	−0.59546	2.5349	0.0029181	17,982	11,901
46	TG_54:6-FA_22:5	−0.59479	2.2304	0.0058829	12,172	8060
47	TG_50:3-FA_14:0	−0.59008	5.5832	0.0000026	198,882	132,119
48	TG_54:7-FA_18:2	−0.58679	3.3337	0.0004638	42,340	28,191
49	TG_46:0-FA_16:0	−0.58611	1.4094	0.0389600	13,465	8969
50	TG_52:3-FA_20:1	−0.58139	1.9335	0.0116550	8352	5582
51	TG_58:9-FA_22:5	−0.57911	2.0937	0.0080598	29,089	19,472
52	TG_50:1-FA_18:0	−0.56879	2.3771	0.0041962	32,625	21,995
53	TG_50:3-FA_18:2	−0.55796	4.7993	0.0000159	235,880	160,224
54	TG_49:1-FA_16:0	−0.47520	2.1050	0.0078522	10,685	7686
55	TG_50:3-FA_18:3	−0.45914	1.6150	0.0242670	7669	5578
56	TG_54:7-FA_18:3	−0.44795	1.6641	0.0216710	31,217	22,885
57	TG_54:1-FA_18:1	−0.43288	1.3638	0.0432680	98,066	72,646
58	TG_58:8-FA_22:5	−0.42954	1.4542	0.0351430	164,362	122,038
59	TG_51:3-FA_18:2	−0.42228	2.2077	0.0061990	32,741	24,433
60	TG_54:6-FA_18:3	−0.41558	2.9443	0.0011369	229,404	171,989
61	TG_54:6-FA_18:2	−0.38200	2.2442	0.0056985	413,911	317,625
62	TG_54:6-FA_18:1	−0.38103	3.4848	0.0003275	190,379	146,190
63	TG_54:7-FA_22:6	−0.36212	1.9103	0.0122950	28,335	22,045
64	TG_56:6-FA_18:3	−0.33875	1.6424	0.0227850	31,784	25,133
65	TG_50:2-FA_18:2	−0.33108	1.9715	0.0106770	168,380	133,852
66	TG_56:4-FA_18:2	−0.32750	1.6495	0.0224150	567,895	452,565
67	TG_54:5-FA_18:0	−0.31409	1.8519	0.0140630	25,947	20,870
68	TG_50:2-FA_18:1	−0.26895	2.2328	0.0058499	794,094	659,037
69	TG_54:5-FA_18:1	−0.25652	2.1656	0.0068302	2,105,658	1,762,655
70	TG_54:5-FA_18:3	−0.25272	2.5468	0.0028392	466,382	391,440
71	TG_54:5-FA_18:2	−0.23562	1.4386	0.0364230	2,519,187	2,139,598
72	TG_56:2-FA_18:0	−0.19089	1.4047	0.0393860	67,576	59,201
73	TG_56:5-FA_18:0	0.19205	1.4961	0.0319040	72,841	83,212
74	TG_52:2-FA_18:1	0.20631	1.7677	0.0170710	8,478,297	9,781,715
75	TG_56:5-FA_20:4	0.27773	1.8963	0.0126980	129,834	157,397
76	TG_52:2-FA_16:0	0.33629	2.4130	0.0038635	4,943,885	6,241,676

**Table 2:** Changes in liver TGs following treatment with seladelpar (Sela) in a HFCC/CDX-induced MASLD model. TG species that showed a significant decrease or increase (negative or positive in the *x*-axis) following Sela administration are listed in order of fold-change using the same ID as in Figure 4. The log_2_ (fold-change), −log_10_ (*p*-value), *p*-value, and mean ppm for 10 mice each of HFCC/CDX (without Sela) and + Sela are shown.

ID	Lipid Species	log_2_ (Fold-Change)	−log_10_ (*p*-Value)	*p*-Value	HFCC/CDX ppm	+Sela ppm
1	TG_52:4-FA_20:4	−0.56836	1.4574	0.0348850	14,467	9755
2	TG_54:5-FA_22:5	−0.44196	1.5848	0.0260130	8599	6331
3	TG_53:4-FA_17:0	−0.41210	1.8692	0.0135130	9989	7508
4	TG_52:4-FA_16:0	−0.29140	1.7570	0.0174980	598,593	489,117
5	TG_52:1-FA_16:0	−0.28171	1.3155	0.0483560	316,852	260,647
6	TG_54:5-FA_18:0	−0.27118	1.4265	0.0374560	25,947	21,501
7	TG_50:2-FA_18:2	−0.24200	1.3143	0.0484960	168,380	142,378
8	TG_52:3-FA_18:2	−0.18542	1.3969	0.0400960	2,664,690	2,343,311
9	TG_52:2-FA_18:1	−0.13583	1.5081	0.0310370	8,478,297	7,716,484
10	TG_53:2-FA_17:0	−0.13193	1.3360	0.0461270	74,596	68,077
11	TG_56:4-FA_20:1	0.23826	1.3921	0.0405390	598,659	706,163
12	TG_58:3-FA_18:1	0.42700	1.4056	0.0393000	136,203	183,117

**Table 3:** Changes in liver TGs following treatment with pioglitazone (Pio) in a HFCC/CDX-induced MASLD model. TG species showing a significant decrease or increase (negative or positive in the *x*-axis) with Pio administration are listed in order of fold-change using the same ID as in Figure 5. The log_2_ (fold-change), –log_10_ (*p*-value), *p*-value, and mean ppm for 10 mice each of HFCC/CDX (without Pio) and + Pio are shown.

ID	Lipid Species	log_2_ (Fold-Change)	–log_10_ (*p*-Value)	*p*-Value	HFCC/CDX ppm	+Pio ppm
1	TG_54:7-FA_16:0	–1.19910	1.8376	0.0145340	3865	1650
2	TG_54:6-FA_22:6	–1.01220	1.9626	0.0108990	30,487	15,115
3	TG_54:5-FA_22:5	–0.85139	2.2843	0.0051965	8599	4766
4	TG_54:7-FA_20:4	–0.68210	1.4253	0.0375550	9253	5767
5	TG_54:6-FA_16:0	–0.54965	1.5095	0.0309380	38,619	26,383
6	TG_51:2-FA_17:1	–0.52185	2.9376	0.0011545	24,085	16,774
7	TG_53:4-FA_17:0	–0.48887	1.3953	0.0402410	9989	7118
8	TG_56:8-FA_16:0	–0.48256	1.4607	0.0346220	25,783	18,453
9	TG_50:3-FA_16:0	–0.47317	1.5569	0.0277370	97,112	69,957
10	TG_56:6-FA_22:5	–0.38571	1.3305	0.0467200	157,853	120,821
11	TG_52:5-FA_16:0	–0.36929	1.4351	0.0367160	54,531	42,214
12	TG_52:4-FA_16:0	–0.36693	2.3219	0.0047652	598,593	464,167
13	TG_54:7-FA_18:2	–0.36481	1.6160	0.0242120	42,340	32,880
14	TG_52:4-FA_18:2	–0.36222	2.2158	0.0060841	1,019,636	793,244
15	TG_56:6-FA_16:0	–0.34514	1.6383	0.0230010	77,342	60,885
16	TG_50:2-FA_16:0	–0.34488	1.9419	0.0114330	615,045	484,268
17	TG_52:5-FA_18:3	–0.31015	1.7551	0.0175760	80,663	65,062
18	TG_48:2-FA_14:0	–0.29404	1.7293	0.0186490	37,639	30,699
19	TG_51:1-FA_16:0	–0.28106	1.3796	0.0417260	16,168	13,306
20	TG_52:3-FA_18:2	–0.27639	2.1055	0.0078425	2,664,690	2,200,113
21	TG_52:3-FA_16:0	–0.25490	2.0389	0.0091427	3,271,335	2,741,534
22	TG_48:1-FA_16:0	–0.24297	1.4033	0.0395100	101,810	86,030
23	TG_52:3-FA_18:1	–0.22231	1.6403	0.0228910	4,006,627	3,434,445
24	TG_52:2-FA_18:1	–0.21913	2.2613	0.0054791	8,478,297	7,283,564
25	TG_50:3-FA_18:2	–0.21455	1.8093	0.0155130	235,880	203,283
26	TG_50:4-FA_18:2	–0.20209	1.3161	0.0482980	51,104	44,425
27	TG_52:2-FA_16:0	–0.19523	1.9825	0.0104110	4,943,885	4,318,141
28	TG_56:5-FA_18:2	0.20265	1.3789	0.0417960	339,646	390,866
29	TG_56:4-FA_18:1	0.27238	1.9126	0.0122280	1,214,492	1,466,862
30	TG_56:3-FA_18:0	0.35739	1.9621	0.0109110	70,215	89,955
31	TG_56:4-FA_20:1	0.38994	2.9712	0.0010686	598,659	784,449
32	TG_56:3-FA_20:1	0.40329	3.0478	0.0008958	782,481	1,034,850
33	TG_56:2-FA_18:0	0.42884	3.1715	0.0006738	67,576	90,968
34	TG_56:4-FA_18:2	0.43182	3.4465	0.0003577	567,895	766,049
35	TG_56:3-FA_18:1	0.43854	3.0589	0.0008731	1,601,137	2,169,912
36	TG_54:1-FA_16:0	0.47499	1.9943	0.0101310	48,439	67,326
37	TG_55:2-FA_18:1	0.49521	1.7544	0.0176050	85,678	120,763
38	TG_56:2-FA_20:1	0.49928	4.8581	0.0000139	58,316	82,428
39	TG_56:2-FA_22:1	0.55618	1.6144	0.0243020	8724	12,825
40	TG_56:2-FA_16:0	0.56189	2.4337	0.0036838	12,981	19,163
41	TG_56:1-FA_22:0	0.58214	1.4346	0.0367610	4951	7414
42	TG_56:2-FA_18:2	0.62138	1.4342	0.0367940	10,641	16,369
43	TG_56:1-FA_18:0	0.62143	2.1817	0.0065815	22,721	34,957
44	TG_56:1-FA_18:1	0.69275	2.0418	0.0090829	19,895	32,155
45	TG_56:2-FA_18:1	0.75507	2.6327	0.0023298	577,543	974,727
46	TG_56:2-FA_20:0	0.80917	2.7715	0.0016924	322,931	565,843
47	TG_56:1-FA_16:0	0.82001	2.8270	0.0014894	6403	11,303
48	TG_56:1-FA_20:0	0.83473	2.6585	0.0021954	19,380	34,562
49	TG_58:3-FA_22:1	0.86349	4.3676	0.0000429	59,155	107,629
50	TG_58:1-FA_18:1	0.86535	2.0327	0.0092753	8731	15,903
51	TG_58:3-FA_18:2	0.88108	2.3936	0.0040401	33,870	62,381
52	TG_58:3-FA_18:1	0.91047	4.4699	0.0000339	136,203	256,015
53	TG_58:3-FA_22:0	0.96267	2.2833	0.0052086	33,025	64,365
54	TG_58:2-FA_22:0	0.98099	2.3157	0.0048334	43,956	86,758
55	TG_58:2-FA_18:1	0.99819	2.6443	0.0022683	90,033	179,840

## Data Availability

The data used to support the findings of this study are available upon request from the corresponding author.

## Supplementary Materials

The following supporting information can be downloaded at https://www.mdpi.com/article/10.3390/biom16081078/s1: Table S1: The formulas and compositions of the CE-2 (standard) and D11061901 (HFCC) diets; Table S2: Fatty acid composition of soybean oil, cocoa butter, and coconut oil from the USDA FoodData Central Food Details; Figure S1: Comparison of the composition ratios of TG molecular species that can constitute TG54:3; Data Source File (a separate Excel file that has four worksheets).

## Abbreviations

The following abbreviations are used in this manuscript:

CDX	Cyclodextrin
FA	Fatty acid
HFCC	High-fat/high-cholesterol/high-cholic acid
HFD	High-fat diet
LC–MS	Liquid chromatography–mass spectrometry
MASLD	Metabolic dysfunction-associated steatotic liver disease
MCD	Methionine/choline-deficient diet
MUFA	Monounsaturated fatty acid
NAFLD	Nonalcoholic fatty liver disease
NASH	Nonalcoholic steatohepatitis
PPAR	Peroxisome proliferator-activated receptor
ppm	parts per million
PUFA	Polyunsaturated fatty acid
SFA	Saturated fatty acid
TG	Triglyceride
