# Supplementary material for: Selective Pharmacological Activation of PPARα/δ/γ Alters the Triglyceride Composition of Fatty Liver in a Diet-Induced MASLD Mouse Model
Source: Biomolecules. 2026 Jul 23;16(8):1078. doi: 10.3390/biom16081078 (PMC13510052; doi:10.3390/biom16081078)
Supplement: Supplementary file 1 [file biomolecules-16-01078-s001.zip › Supplementary Materials.pdf]

Supplementary Materials

Selective Pharmacological Activation of PPARα/δ/γ Alters the Triglyceride Composition of Fatty Liver in a Diet-Induced MASLD Mouse Model

Takako Hishiki [1,†], Akihiro Honda [1,†], Reina Tanaka [1], Yukiko Minomo [1], Otoe Minamisawa [1], Tsubasa Konno [1], Shotaro Kamata [1], Waka Kamichatani [1], Suzumi M. Tokuoka [2], Yoshihiro Kita [2], Hideo Shindou [3,4] and Isao Ishii [1,*]

[1] Department of Health Chemistry, Showa Pharmaceutical University, Machida, Tokyo 194-8543, Japan
[2] Department of Lipidomics, Graduate School of Medicine, The University of Tokyo, Bunkyo, Tokyo 113-0033, Japan
[3] Department of Lipid Life Science, Japan Institute for Health Security, Shinjuku, Tokyo 162-8655, Japan
[4] Department of Medical Lipid Science, Graduate School of Medicine, The University of Tokyo, Bunkyo, Tokyo 113-0033, Japan
* Correspondence: i-ishii@ac.shoyaku.ac.jp
† These authors contributed equally to this work.

Contents

1. Table S1. The formulas and compositions of the CE-2 (standard) and D11061901 (HFCC) diets.
2. Table S2. Fatty acid composition of soybean oil, cocoa butter, and coconut oil from the USDA FoodData Central Food Details.
3. Figure S1. Comparison of the composition ratios of TG molecular species that can constitute TG54:3.
4. Data Source File (a separate Excel file that has four worksheets)

Table S1. The formulas and compositions of the CE-2 (standard) and D11061901 (HFCC) diets.

Product	**CE-2** (Standard diet, CLEA Japan)		**D11061901** (HFCC diet, Research Diet)		
Formula:				gram	%
	Soybean lees/oil	unknown	Casein	75	8.759
	Whitefish meal	unknown	Soy protein	130	15.182
	Yeast	unknown	DL-Methionine	2	0.234
	Embryo bud	unknown	Corn starch	16	1.869
	Alfalfa	unknown	Maltodextrin	150	17.51
	Flour	unknown	Sucrose	30	3.503
	Vitamins & minerals	unknown	Cellulose, BW200	90	10.510
	Others	unknown	Soybean oil	86	10.043
	Cocoa butter	1.00%	Cocoa butter	129	15.065
	(Formula unpublished)		Coconut oil, 76	60.2	7.030
			Mineral mix S10001	35	4.087
			Calcium carbonate	5.5	0.642
			Sodium chloride	8	0.934
			Potassium citrate	10	1.168
			Vitamin mix V10001	10	1.168
			Choline bitartrate	2	0.234
			Cholesterol	12.5	1.460
			Sodium cholic acid	5	0.584
			FD&C Blue Dye #1	0.1	0.012
			Total	856.3	100.00
Basic composition:	2025 average (CLEA Japan web site)				
	Moisture (%)	7.51		unknown	
	Crude protein (%)	24.85		24 (20 kcal%)	
	Carbohydrate (%)	unknown		24 (20 kcal%)	
	Crude fat (%)	4.33		32 (60 kcal%)	
	Crude fiber (%)	4.61		unknown	
	Crude ash (%)	7.14		unknown	
	Nitrogen free extract (%)	51.57		unknown	
	Calories (kcal/g)	3.446		4.8	

The composition of CE-2 (mean values in 2025) is published on the CLEA Japan website: https://www.clea-japan.com/en/ (accessed June 15, 2026).

Table S2. Fatty acid composition of soybean oil, cocoa butter, and coconut oil from the USDA FoodData Central Food Details (accessed on 1 June 2026).

Product: NDB Number	**Oil, soybean: 4044**	**Oil, cocoa butter: 4501**	**Oil, coconut: 4047**
Formula: portion 100 g	Total fat (NLEA) 94.6 g	Total lipid (fat) 100 g	Total lipid (fat) 99.1 g
**Total saturated fatty acid (SFAs) (g)**	**14.9**	**59.7**	**82.5**
SFA 4:0 (butyric acid)	not tested	0	0.009
SFA 6:0 (caproic acid)	not tested	0	0.477
SFA 8:0 (caprylic acid)	not tested	0	6.8
SFA 10:0 (capric acid)	not tested	0	5.39
SFA 12:0 (lauric acid)	not tested	0	41.8
SFA 14:0 (myristic acid)	0.075	0.1	16.7
SFA 15:0 (pentadecanoic acid)	0.014	not tested	0.015
SFA 16:0 (palmitic acid)	10.3	25.4	8.64
SFA 17:0 (margaric acid)	0.086	not tested	0.009
SFA 18:0 (stearic acid)	3.71	33.2	2.52
SFA 20:0 (arachidic acid)	0.294	not tested	0.076
SFA 21:0 (heneicosylic acid)	0.025	not tested	not tested
SFA 22:0 (behenic acid)	0.306	not tested	0.019
SFA 24:0 (lignoceric acid)	0.124	not tested	0.031
**Total monounsaturated fatty acids (MUFAs) (g)**	**22.1**	**32.9**	**6.33**
MUFA 14:1 (myristoleic acid)	not tested	not tested	0
MUFA 15:1 (pentadecenoic acid)	not tested	not tested	0
MUFA 16:1 (palmitoleic acid)	0.08	0.2	0.016
MUFA 17:1 (heptadecenoic acid)	not tested	not tested	0
MUFA 18:1 (oleic acid)	21.4	32.6	6.27
MUFA 20:1 (eicosenoic acid)	0.6	0	0.041
MUFA 22:1 (docosenoic acid)	not tested	0	0
MUFA 24:1 (tetracosenoic acid)	not tested	not tested	0
**Total polyunsaturated fatty acids (PUFAs) (g)**	**57.6**	**3**	**1.7**
PUFA 18:2 n-6 c,c (linoleic acid)	50.9	2.8	1.68
PUFA 18:3 (octadecatrienoic acid)		0.1	
PUFA 18:3 n-3 c,c,c (α-linolenic acid)	6.62	not tested	0.019
PUFA 18:3 n-6 c,c,c (γ-linolenic acid)	not tested	not tested	0
PUFA 18:4 (parinaric acid)	not tested	0	0
PUFA 20:2 n-6 c,c (eicosadienoic acid)	not tested	not tested	0
PUFA 20:3 n-3 (icosatrienoic acid)	not tested	not tested	0
PUFA 20:3 n-6 (eicosatrienoic acid)	0	not tested	0
PUFA 20:4 (icosatetraenoic acid)	not tested	0	0
PUFA 20:5 n-3 (eicosapentaenoic acid: EPA)	not tested	0	0
PUFA 22:4 (docosatetraenoic acid)	not tested	not tested	0
PUFA 22:5 n-3 (docosapentaenoic acid: DPA)	not tested	0	0
PUFA 22:6 n-3 (docosahexaenoic acid: DHA)	not tested	0	0
**Total trans fatty acids (g)**	not tested	not tested	0.028

USDA, United States Department of Agriculture; NLEA, Nutrition Labeling and Education Act. The four-digit numbers is web links to USDA FoodData Central Food Details.

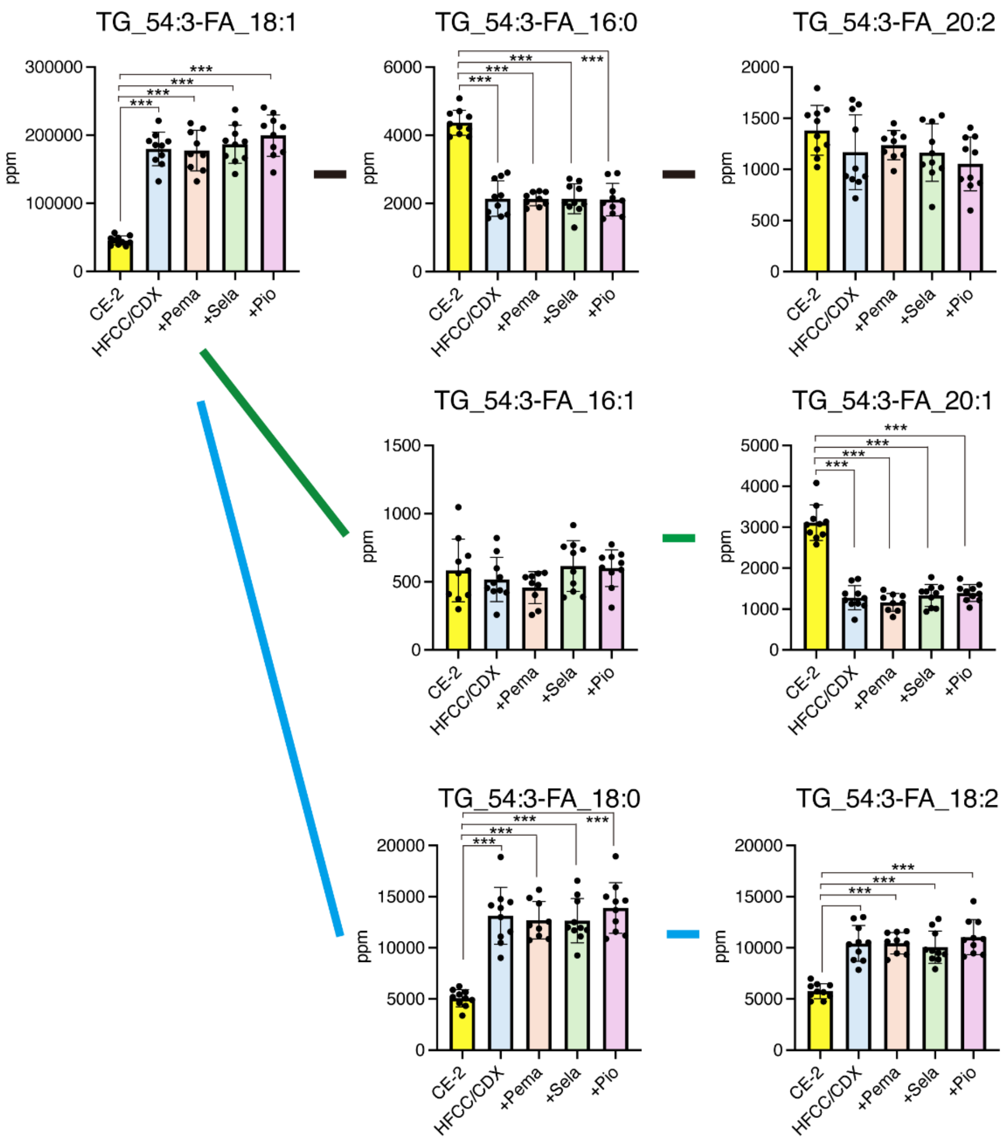

Figure S1. Comparison of the composition ratios of TG species that can constitute TG54:3. The detected TG54:3_FA18:1 could potentially be one of four TG molecular species: TG54:3_FA18:1×3, TG54:3_FA18:1-FA16:0-FA20:2, TG54:3_FA18:1-FA16:1-FA20:1, or TG54:3_FA18:1-FA18:0-FA18:2. The detected TG composition patterns suggest that TG54:3_FA18:1×3 is the most abundant, followed by TG54:3_FA18:1-FA18:0-FA18:2, with the others present in small amounts. Data are mean ± SD (n = 9 for Pema and 10 for others) and included in the Data Source File. A one-way ANOVA using Tukey's multiple comparison test revealed a statistically significant difference at ***p<0.001.
